# Energy Band Gap Investigation of Biomaterials: A Comprehensive Material Approach for Biocompatibility of Medical Electronic Devices

**DOI:** 10.3390/mi11010105

**Published:** 2020-01-18

**Authors:** Ashkan Shafiee, Elham Ghadiri, Jareer Kassis, David Williams, Anthony Atala

**Affiliations:** 1 Wake Forest Institute for Regenerative Medicine, Wake Forest School of Medicine, Winston-Salem, NC 27101, USA; 2 Department of Chemistry, Wake Forest University, Winston-Salem, NC 27109, USA; 3 Comprehensive Cancer Center, Wake Forest School of Medicine, Winston-Salem, NC 27101, USA

**Keywords:** energy band gap, optical absorption, biomaterial, bio-optoelectronics, advanced biomanufacturing

## Abstract

Over the past ten years, tissue engineering has witnessed significant technological and scientific advancements. Progress in both stem cell science and additive manufacturing have established new horizons in research and are poised to bring improvements in healthcare closer to reality. However, more sophisticated indications such as the scale-up fabrication of biological structures (e.g., human tissues and organs) still require standardization. To that end, biocompatible electronics may be helpful in the biofabrication process. Here, we report the results of our systematic exploration to seek biocompatible/degradable functional electronic materials that could be used for electronic device fabrications. We investigated the electronic properties of various biomaterials in terms of energy diagrams, and the energy band gaps of such materials were obtained using optical absorption spectroscopy. The main component of an electronic device is manufactured with semiconductor materials (i.e., E_g_ between 1 to 2.5 eV). Most biomaterials showed an optical absorption edge greater than 2.5 eV. For example, fibrinogen, glycerol, and gelatin showed values of 3.54, 3.02, and 3.0 eV, respectively. Meanwhile, a few materials used in the tissue engineering field were found to be semiconductors, such as the phenol red in cell culture media (1.96 eV energy band gap). The data from this research may be used to fabricate biocompatible/degradable electronic devices for medical applications.

## 1. Introduction

**Figure 1:**
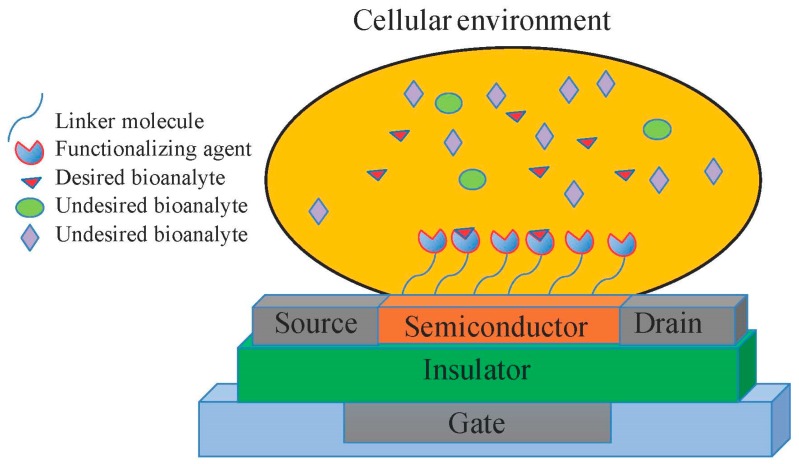
A transistor-based biosensor. An example of a medical electronic device where semiconductor materials interact directly with cellular environment. In this case, functionalizing agents for a desired bioanalyte are attached to the surface of the transistors using appropriate linker molecules. The active layer of the transistor including the semiconductor materials are in contact with cellular environment to real-time monitor and control the environment. The selection of correct functionalizing agent may lead to high selectivity of the device by detecting only the desired bioanalyte. Upon detection of the bioanalyte, the electronic characterizations of the active layer may change and consequently the amount of target bioanalyte is related to the variation in transistor performance (i.e., current-voltage graph). The interaction between the semiconductor layer and cellular environment necessitates the use of biocompatible materials to enable the use of the medical electronic device for cells and tissues.

Tissue engineering uses physical and biological sciences, as well as biomedical technology to enhance human life by providing human tissues and eventually organs [1,2]. Advancements in tissue engineering, based on its underlying science [3] and technology [4], have opened new avenues for clinical applications [5]. Both scaffold-free and scaffold-based tissue engineering techniques have demonstrated the capability to enhance medical treatment [6,7,8,9,10,11]. Despite major advances in the field, many drawbacks of using current technologies remain unaddressed. Real-time monitoring of tissue development may enable tissues to achieve functional control at the cellular level [12]. Such monitoring may also be important after organ transplantation [13]. It has been shown that one of five patients on the U.S. organ transplantation list who have previously received a transplanted organ lost it owing to chronic rejection by the immune system [14]. Real-time monitoring systems could enable physicians to diagnose such events early, leading to prompt intervention. Moreover, advanced biomanufacturing involves many steps, including a monumental cell culturing effort [15]. An automated, fully monitored cell culture system consisting of biosensors for metabolism-generated bioanalytes and other essential bioactive factors can enhance the cell culture quality and reduce costs by optimizing the usage of expensive cell maintenance materials [16]. Such real-time monitoring and controlling technologies necessitate tissue engineers to employ electronic devices compatible with cells and tissues. For example, transistor-based biosensors and drug release systems are electronic devices that may interact with cells. Transistors are one of the most important electronic components of electronic circuits, and the fabrication of such devices may enhance automatic tissue monitoring and controlling. As shown in Figure 1, a transistor encompasses an insulator layer as well as the source, drain, and gate that are normally made of conductive materials such as metals. The main part of a transistor, however, is the semiconductor material, the invention and application of which was a revolution in the electronic devices industry. In transistor-based biosensors, the active layer of the device interacts with the cellular environment, and the detection of a bioanalyte would change the resistivity or conductivity of the layer causing an electronic response such as a detection pulse. Therefore, semiconductor materials that can be adjacent to the cells are important for reliable medical electronic device fabrication. Figure 1 shows a transistor-based biosensor and its components.

Many technologies have been used to fabricate electronic devices. Among them, printing technologies have become popular over the last decade [17,18,19]. Printed electronics hold great promise in terms of fabricating low-cost, flexible, and portable electronic devices such as transistors [20], power cells [21,22], and biosensors [23]. Perhaps the most critical task of fabricating electronic devices for medical applications is identifying the required electronic materials [24]. To that end, the only approach that has been employed to search for electronic materials is to test for the biocompatibility of known electronic materials [25].

Organic materials such as poly (3-hexylthiophene-2,5-diyl) (P3HT) and poly (3,4-ethylenedioxythiophene)-poly (styrenesulfonate) have been tested in the presence of different cells to assess their biocompatibility [26,27]. This approach is prone to two potential drawbacks. First, these biocompatibility assessments might be incomplete; as “biocompatibility” is not the property of the material alone [28] but is rather a property of both the material and biological systems together. Therefore, any material must be tested for compatibility with each cell type. Hence, many investigations concentrate on particular aspects of biocompatibility such as cell adhesion, owing to the fact that assessing electronic materials with different cell types may be time consuming and costly [26]. Second, many functional electronic materials are found not to be biocompatible, which limits their use in electronic device fabrication for medical applications. 

A potentially productive approach would be to explore the electronic properties of biomaterials that are regularly used for cell culture, and tissue engineering; as their biocompatibility has already been defined. Although this method would bring numerous new potential material candidates for biocompatible electronics, there are no reports of such an approach to our knowledge. We previously demonstrated inkjet printing of a reactive species scavenger that is also used for optoelectronics [29]. To achieve a comprehensive material approach for medical electronics, we introduce a novel and systematic approach towards searching for functional electronic materials that can work adjacent to cells and tissues. Electronic devices such as transistor-based biosensors include three classes of materials: insulators, semiconductors, and conductors. These three categories of materials are distinguished by their E_g_ and energy band diagram. Materials with E_g_ values lower than 1 eV and greater than 2.5 eV are considered conductors and insulators, respectively. The most important part of the electronic devices, however, are semiconductors with E_g_ values between 1 and 2.5 eV. Therefore, we focused on materials within this E_g_ range. 

## 2. Materials and Methods

We used optical spectroscopy to obtain the energy band gap (E_g_), which is defined as the amount of energy that one electron requires to transport from the valence band to the conduction band. In molecules, this E_g_ is the difference in energy between the highest occupied molecular orbital (HOMO) and lowest unoccupied molecular orbital (LUMO). The E_g_ is used to divide materials into three different categories: insulators, semiconductors, and conductors. We previously used this technique to obtain the energy band diagram of organic functional electronic materials for optoelectronic applications [30]. Here we apply the same technique on biomaterials with unknown electronic properties to identify semiconductor biomaterials for biocompatible device fabrication.

**Figure 2:**
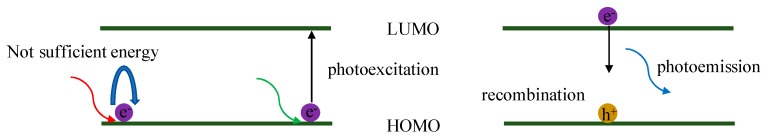
Energy band diagram, photoexcitation, and photoemission. (**Left panel**): Photons with low energy (large wavelengths) cannot excite an electron, whereas photons with higher energies (shorter wavelengths) can. Upon photoexcitation, electrons are transferred from the highest occupied molecular orbital (HOMO) to lowest unoccupied molecular orbital (LUMO) leaving holes in the HOMO. (**Right panel**): If no electronic interactions occur during the very short lifetime of an electron in the LUMO, it returns to the HOMO and emits a photon (radiative electron-hole recombination).

In optical absorption spectroscopy, several packets of photons are illuminated on a material, and the transmitted light is collected using a detector. For this procedure, each photon carries a defined amount of energy based on the wavelength (or color in visible light) equal to:

(1) E=hν=hC/λ

where *h*, *C*, *v*, and *λ* are Planck’s constant (6.63 × 10^−34^ *JS*), speed of light (299,792,458 ms^−1^), frequency (Hz), and wavelength (nm) of the photon, respectively. Contrary to classical mechanics where an object has a continuous range of energies, the allowed energies for electrons in a material in quantum mechanics are constrained to defined discrete levels. Provided that the photon delivers at least the E_g_, an electron can become excited and absorb the energy of the photon and change its discrete level of energy from HOMO to LUMO. Therefore, if the photon’s energy is less than the E_g_ required to transport from HOMO to LUMO, it cannot be absorbed by the electron, and the spectrometer would detect it as a transmitted photon. Figure 2 shows the absorption of a photon with sufficient energy to excite an electron as well as photoemission based on electron recombination.

By scanning the entire visible range of the electromagnetic spectrum and measuring the optical transmission, we could identify the absorption edge or absorption peak that corresponded to the absorption of photons with sufficient energy to excite an electron from HOMO to LUMO. Considering the fact that we are mostly interested in discovering semiconductor materials with an E_g_ between 1 and 2.5 eV, we calculate the appropriate wavelength to be 495–1200 nm (Equation (1)). Therefore, these materials that show evidence of a rise in the absorption spectrum within this wavelength range can be considered potential semiconductors. This approach can help to investigate the vast majority of biomaterials to be potentially used for biocompatible electronic device fabrication. We used a UV-Vis Lambda 950 spectrometer (Perkin-Elmer, Waltham, MA) with integrating spheres for opaque samples (Labsphere, North Sutton, NH). Various biomaterials including fibrinogen, glycerol, Dulbecco’s modified eagle medium (DMEM), phenol red-free DMEM, and gelatin were used to obtain the optical absorption spectra using 3 mL of materials in a quartz cuvette (PerkinElmer, Waltham, MA, USA). The absorption spectra were obtained by sweeping wavelengths from 900 to 250 nm. The respective wavelengths in the cross point between the onset of the spectra and the baseline were used to calculate the E_g_ using Equation (1). It is worth noting that an insulator that is colored can cause a false positive reading as a semiconductor. Therefore, after the absorption experiment, one needs to confirm that the material has an electronic interaction; i.e., that the rise in absorption is solely produced by the excited electrons, not just the effect of color of the material (such as structural colors). Furthermore, it is important to determine whether the semiconductor exhibits appropriate electronic interactions. We investigated whether the photoluminescence (PL) of the semiconductor biomaterials as well as PL quenching at the interface differed among biocompatible semiconductors in multi-component samples. This was performed using a bilayer of the biomaterial of choice and a known P3HT polymer with an LSM 880 confocal microscope (Carl Zeiss, Germany). Glass substrates were washed using an ultrasonic bath with Micro 90, ethanol, and acetone (15 min each) rinsed with distilled water and dried with nitrogen. Before deposition, the substrates were plasma-cleaned with high power for 45 s. P3HT was drop-casted onto the glass substrates, and DMEM was deposited on the samples and tested for PL quenching.

## 3. Results and Discussion

The optical absorption spectra of several biocompatible materials were investigated to obtain the E_g_. Glycerol, hyaluronic acid, gelatin, fibrinogen, and DMEM are all heavily used in cell culturing, tissue engineering and regenerative medicine [31,32,33]; hence, we examined their potential as semiconductors. 

**Figure 3:**
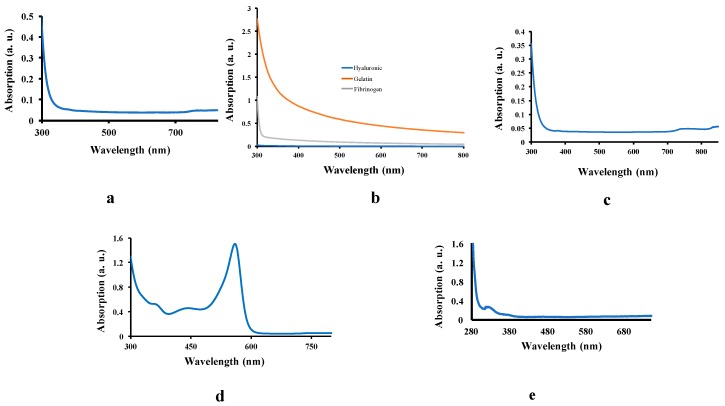
Absorption spectra of different materials (**a**) glycerol, (**b**) hyaluronic acid-gelatin-fibrinogen, (**c**) a closer look at hyaluronic acid, (**d**) the absorption spectrum of cell culture medium, Dulbecco’s modified eagle medium (DMEM). The rise in the absorption starts at 630 nm (equal to 1.96 eV). This means that photons with larger wavelengths (i.e., lower energies) cannot provide sufficient energy to excite electrons. (**e**) the absorption spectrum for phenol red-free DMEM does not show any rise above 400 nm.

The increase in the absorption spectrum indirectly demonstrated an increasing number of absorbed photons and therefore, excited electrons. The E_g_ of a material is obtained by the wavelength (energy) of the first photon that is absorbed, and causes a rise in the absorption spectrum. As shown in Figure 3a–c, the absorption spectra of biomaterials such as glycerol, hyaluronic acid, gelatin, and fibrinogen do not show a peak or a rise above 400 nm. In order to identify a material as a small bandgap semiconductor, the E_g_ should be between 1 and 2.5 eV, which corresponds to a photon wavelength of 495–1200 nm (visible-near infrared). In Figure 3b, for example, the gelatin shows a higher level of absorption as well as an increase to approximately 3 eV. However, for gelatin, there is no clear peak in the visible region of the spectra. The flat baseline is at approximately 0.05 in our measurements, as shown in the data presented in panels a, b (for hyaluronic acid), c, d, and e. In panel b, the fibrinogen and gelatin samples show some deviation from the baseline; this monotonous absorption is caused by optical scattering effects in the relatively turbid fibrinogen sample and more turbid gelatin sample. The origin of such a pattern in the absorption spectrum is caused by optical loss following optical scattering and not by electronic absorption (as is the case in semiconductors that are characterized by electronic absorption features, absorption peaks, or absorption edges). Notably, the rises in the absorption spectrum and peak provide some meaningful information about the electronic interaction. The absorption edges of fibrinogen and glycerol were 3.54 and 3.02 eV, respectively. The DMEM cell culture medium shows a clear peak at approximately 550 nm and a rise at 630 nm; the latter demonstrates an energy band gap (increasing number of electrons that possess sufficient energy to be excited). Using Equation (1), the 630 nm is equal to 1.96 eV that falls into the semiconductor E_g_ range. Therefore, the cell culture medium can be considered a potential semiconductor. It is worth noting that, among the components of DMEM, it is phenol red that provides this absorption and must be tested for electronic interactions before confirmation as a semiconductor. Figure 3e shows absorption spectrum of phenol red-free DMEM confirming that the rise and peak in 3d are caused by phenol red. 

## 4. Electronic Interaction Verification

**Figure 4:**
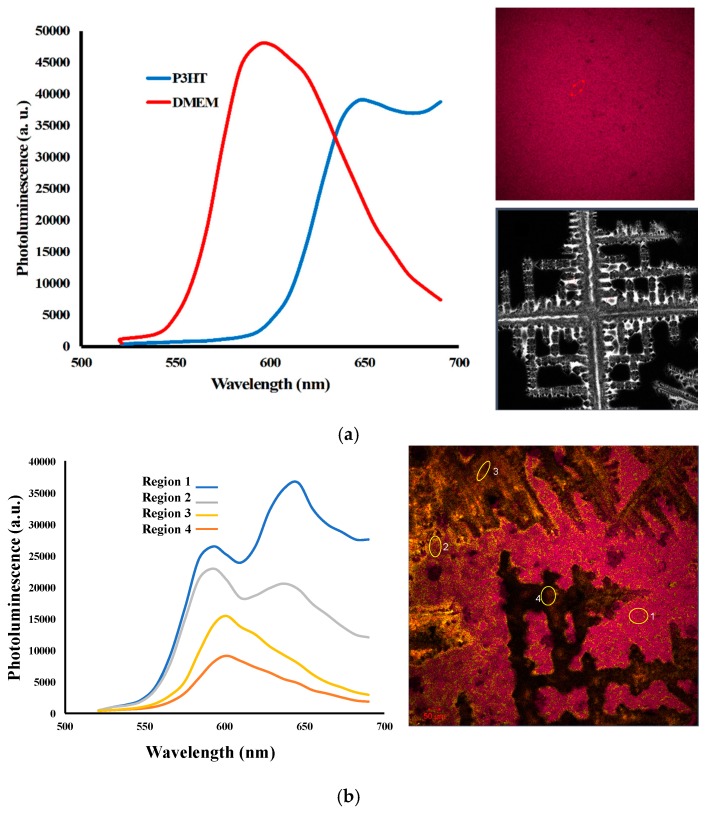
(**a**) Right top: A single layer film of a known P-type organic material (i.e., P3HT) and Right below: Confocal Microscopy of DMEM Left: PL of P3HT and DMEM. (**b**) Photoluminescence of different regions in a bilayer film with different properties. Region 1 has more P3HT compared to DMEM. It shows peaks in ~580 and 640 nm representing phenol red and P3HT, respectively. Region 2 has less P3HT compare to region 1, but the two peaks are still clearly observable. The peak is quenched in regions 3 and 4 that confirm the electronic interaction between two materials. It is clear that the peak for phenol red also has much less intensity for regions 3 and 4 compared to regions 1 and 2. The quenching procedure confirms an electronic interaction between two materials in the bilayer film that reduces the number of recombinations in electrons/holes and emitted photons. The photoluminescence spectra of different regions show partial to complete quenching for P3HT peak. The peak caused by DMEM is also quenched partially.

The absorption experiment can provide a quantitative measurement of the E_g_ based on the wavelength of the first photon that has sufficient energy to excite an electron from the HUMO to the LUMO. However, as described above, a transparent color object can produce the same optical spectroscopy results and can be thought to have an appropriate E_g_. Therefore, an absorption experiment is necessary but insufficient for discovering semiconductor biomaterials. As such, we conducted PL spectroscopy for a bilayer film of the biomaterial and a known organic polymer, i.e., P3HT. In theory, the time that an excited electron can spend on the LUMO is very short based on the Heisenberg uncertainty principle. This is demonstrated with a simple calculation:

(2) $$\sigma_{E}\sigma_{t}\geq \hbar /2$$

where *σ_E_*, *σ_t_* are the expectation value of the energy operator in the state *ψ* and the lifetime of the state *ψ*, respectively. This indicates that excited states like the LUMO would have extremely short lifetimes. As such, if there is no electronic interaction to remove an excited electron from the LUMO, it returns to the HOMO and emits a photon. This recombination can be detected using PL that measures the number of emitted photons (Figure 2). Therefore, the peak in the PL graph implies a high number of electrons that move back to the HOMO and recombine with holes. However, if the electronic system includes both electron donor and acceptor materials (p- and n-type materials), the excited electron can be transferred to the acceptor. This electronic transfer would decrease the number of recombinations and quench the PL spectrum. Therefore, using the PL measurements of the material system in conjugation with a known polymer, one can verify if the material shows such electronic interactions (charge separation). Here, we used P3HT, a well-known p-type organic material that is used as an electron donor in electronic device fabrication. Such systems are modeled as donor-acceptor heterojunctions. Depending at the energy levels of the HOMO and LUMO of the two materials, the electrons and holes become separated; electrons fall into lower energy states and holes rise to higher energies [34,35]. Such charge separation decreases the radiative recombination. The heterogeneous charge transfer and PL quenching has been observed in a variety of organic or inorganic semiconductor systems applied for optoelectronics and solar energy conversion systems [36,37]. We investigated the optoelectronic properties of the biomaterials in our current study. Therefore, the observed PL quenching in our experiment is attributed to interfacial charge separation at the phenol red-P3HT junction. In the scenario that both electrons and holes are injected into the same material (provided that the relative energy levels of the two materials allow such charge transfer), the concentrations of the carriers in one material can increase, resulting in higher PL. This is similar to the process that occurs in quantum well-based light-emitting diodes. Figure 4 shows the confocal PL mapping and PL spectra of single-layer P3HT as well as a bilayer film of P3HT and biomaterial. The PL spectrum of the P3HT layer shows a characteristic peak at 630 nm. The PL map also shows uniform photoemission over a large area (scan area 1.4 × 1.4 mm^2^). In the bilayer film of medium/polymer, four different regions were investigated to verify the effect of electronic interactions. Figure 4a shows the confocal images of P3HT and DMEM as well as their PL spectra. In Figure 4b, the PL spectra for regions 1 and 2 show peaks at 580 and 630 nm representing phenol red and P3HT, respectively. As shown in Figure 4b, the amount of medium in region 2 is higher than that in region 1; therefore, the peaks for both phenol red and P3HT are smaller in region 2 owing to more electronic interactions among molecules. It is worth noting that the medium and polymer interaction in regions 3 and 4 can quench the peak for P3HT completely. The quench in the PL spectrum for bilayer film confirms the electronic interaction between the P3HT and biomaterial. With the charge separation between the biomaterial and P3HT; the number of recombinant electrons in the P3HT drops, and the PL spectrum thereby quenches. 

Any biomaterial can be tested using these experiments to investigate their electronic properties. As revealed in the absorption experiment, the E_g_ values of several biomaterials are quite sizable. However, DMEM (particularly the phenol red component) can be considered a semiconductor, which can be used for further device fabrication.

## 5. Conclusions

We employed a novel technique to investigate the electronic properties of biomaterials using energy band diagram studies. Several biomaterials have been tested to obtain optical band gaps that represent the difference between the HOMO and LUMO energy levels. We discovered that the cell culture medium DMEM has an E_g_ equal to 1.96 eV. To verify the effect of electronic interaction, we conducted PL spectrometry using a bilayer of desired biomaterial and a known organic polymer such as P3HT. The quenching in PL confirmed the electronic interactions between the two materials. This material study approach can introduce new avenues of research to discover more biocompatible semiconductors essential for biocompatible/degradable electronic fabrication. In addition to elucidating the biocompatibility of functional electronic materials that are globally investigated, we envision a comprehensive material approach for successful device fabrication by testing the electronic properties of biomaterials.
